# Effect of Waste Glass Powder Replacement of Hydraulic Lime on Properties of Natural Hydraulic Lime Mortars

**DOI:** 10.3390/ma17215247

**Published:** 2024-10-28

**Authors:** Murat Sahin, Polat Ozyigit

**Affiliations:** 1 School of Civil Engineering, University College Dublin, D04 V1W8 Dublin, Ireland; 2 Department of Civil Engineering, Yozgat Bozok University, 66900 Yozgat, Türkiye; polat.ozyigit@bozok.edu.tr

**Keywords:** natural hydraulic lime, glass powder, mortar, carbonation, mechanical properties

## Abstract

This paper investigates the effects of the partial replacement of natural hydraulic lime (NHL) with waste glass powder (GP) on the physical, mechanical, and microstructural properties of NHL mortars. In the experimental study, five mixtures containing up to 50% GP were prepared to evaluate its effect on the flow, carbonation, unit weight, water absorption, porosity, ultrasonic pulse velocity, capillary water absorption, compressive strength, and microstructure of NHL mortars. The experimental results suggest that the partial replacement of NHL with GP significantly affects the properties of NHL mortars. A reduction in compressive strength was observed with increasing GP content in mortars at both early and later stages. Nevertheless, the compressive strength difference between samples containing 50% GP and the reference was found to be relatively minor at 91 days, implying an enhanced pozzolanic reaction over time. The incorporation of GP improved the consistency and capillary water absorption of mortars, while the opposite was observed for ultrasonic pulse velocity, porosity, and water absorption. The microstructural analysis revealed distinct changes in the structure of samples incorporating GP. The partial substitution of hydraulic lime with GP could be beneficial in reducing the CO_2_ emissions of NHL mortars.

## 1. Introduction

Portland cement, the main binder used in the construction industry with a global production of about four billion tons per year, has become a growing concern owing to its environmental impact [1,2,3]. The production process of Portland cement releases significant amounts of CO_2_, accounting for over 8% of anthropogenic CO_2_ emissions [4]. Additionally, Portland cement is not suitable for the restoration or repair of historic buildings owing to its incompatible properties, including low vapour permeability, high thermal expansion, high compressive strength, and high soluble salts content [5,6]. Due to the above-mentioned facts, lime-based binders are a feasible alternative that has attracted the interest of researchers in conservation and restoration works [7].

Natural hydraulic lime (NHL), which is obtained from the calcination of limestones containing clay or silica, possesses the ability to set and harden through a reaction with water and carbon dioxide from the atmosphere [8]. NHLs have calcination temperatures of 950–1250 °C [9]. Thus, the production process of NHLs emits a considerable amount of CO_2_, but not as much as Portland cements, which have calcination temperatures of about 1450 °C [10]. Today, researchers have been seeking alternative methods not only to reduce environmental impact but also to improve the properties of lime-based binders [11]. One of these is the utilisation of mineral additives referred to as cement replacement materials, supplementary cementitious materials, or pozzolans with NHL [12]. Hence, there has recently been a growing interest in the utilisation of supplementary cementitious materials such as fly ash [13,14], metakaolin [15,16,17,18], silica fume [19,20,21,22], various types of slags [22,23,24,25], or various combinations of pozzolans [26,27] in NHL mortars. On the other hand, waste glass powder, which is a widely used as a replacement material in cement-based composites, has been the subject of a limited number of research with lime-based composites.

Glass is a conventional material with a broad range of applications owing to its remarkable properties, including optical transparency, chemical inertness, high intrinsic strength, and low permeability [28]. It is generally considered a fully recyclable material, in principle; however, there are certain limitations that can affect the recycling process [29]. Global glass production was estimated to be about 77 million tons in 2014, of which 36% was recycled and the remaining was landfilled [30]. Therefore, the utilisation of waste glass is of great importance in terms of the conservation of natural resources and the reduction in carbon footprint. Previous studies have identified the potential use of waste glass as an aggregate, mineral additive, binder, or raw material for cement production in the construction industry [30,31,32,33,34]. There is a lot research on the use of glass powder as a cement replacement material, or supplementary cementitious material, examining the behaviour of cement and glass powder with/without various additives [35,36,37,38,39,40,41,42]. Additionally, it is possible to utilise glass powder as binder or activator in alkali-activated materials [43,44,45,46]. In consideration of the given literature above, the use of glass powder with hydraulic lime is a feasible option owing to its pozzolanic properties. However, there is a limited number of studies examining glass powder incorporated in hydraulic lime-based composites. Edwards et al. [47] replaced natural hydraulic lime with glass powder up to 75% by weight to assess the effect of glass powder on the mechanical, chemical, and microstructure of mortars. It was found that substituting hydraulic lime with 10% glass powder resulted in increased compressive strength; however, such improvements were not observed at higher contents of substitutions. In addition, the findings of the study provided grounds for the pozzolanic effect of glass powder in hydraulic lime mortars. Fragata et al. [48] compared the mechanical properties of cement, lime, and hydraulic mortars containing glass powder. It was reported that the addition of glass powder to hydraulic lime mortars acts as a pozzolan, leading to enhanced mechanical strength. Vyšvařil et al. [49] investigated the properties of hydraulic lime-based mortars containing foam glass powder which is composed of about 95% of glass. They incorporated foam glass powder up to 40% in hydraulic lime mortars. They found slight improvements in the mechanical properties at early ages of hydraulic lime mortars with the increase in foam glass powder content, but similar behaviour was not observed at later ages.

This paper investigates the effect of the partial replacement of natural hydraulic lime (NHL) with glass powder on the physical, mechanical, and microstructural properties of NHL-based mortars. Therefore, five mixtures with glass powder contents up to 50% were prepared and tested in order to evaluate the effect of glass powder on the flow, carbonation, unit weight, water absorption, porosity, ultrasonic pulse velocity, capillary water absorption, compressive strength, and microstructure of NHL-based mortars.

## 2. Materials and Methods

### 2.1. Materials

**Figure 1:**
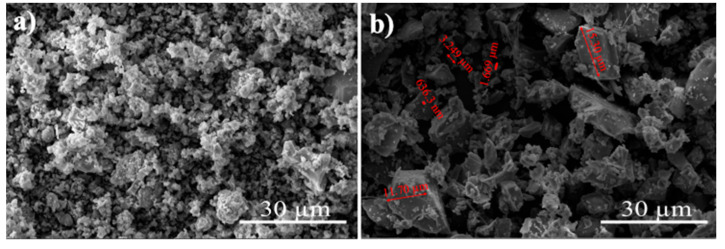
SEM images: (**a**) natural hydraulic lime, (**b**) glass powder.

The materials used in this study were natural hydraulic lime (NHL), glass powder (GP), silica sand, and tap water. The type of natural hydraulic lime was NHL3.5, complying with EN 459-1 [8]. The density and fineness of the powders were determined using Le Chatelier flask and Blaine apparatus in accordance with EN 196-6 [50], respectively. The density and Blaine fineness of NHL were 2556 kg/m^3^ and 8950 cm^2^/g, respectively. Glass powder (GP) was obtained by grinding waste soda-lime green bottles in a ball mill. First, the bottles were kept in hot water for one hour for the removal of the label. Then, the bottles were cleaned and dried in the oven at 105 °C for 24 h prior to ball milling. The diameter of the ball mill chamber was 200 mm. A combination of 48 mm and 15 mm diameter of sphere steel balls in size were used as grinding media. The ratio of grinding media to glass bottles was kept at 7/1 by mass. Five bottles were introduced to the chamber and ground for 3 h. The density and Blaine fineness of the glass powder determined was 2574 kg/m^3^ and 3560 cm^2^/g, respectively. The oxide composition of powders was determined through X-ray fluorescence (XRF) analysis and is provided in Table 1. The scanning electron microscope (SEM) image of NHL and GP at the same magnification and scale are given in Figure 1. As seen from the figure, the particle sizes of NHL are quite finer than those of GP. Moreover, most of the glass powder particles are more angular and smoother than those of hydraulic lime particles.

**Figure 2:**
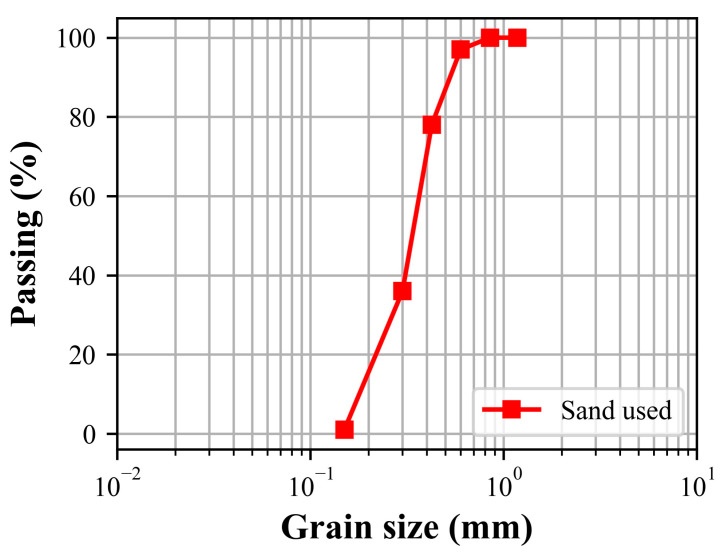
Particle size distribution of the sand used.

**Figure 3:**
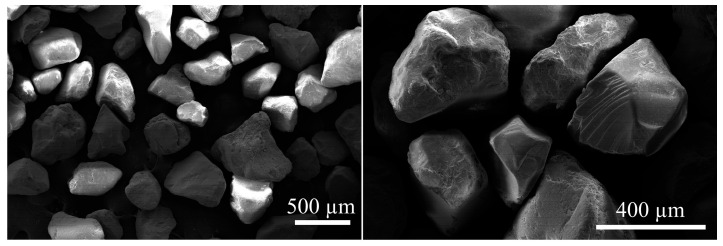
SEM images of the silica sand used.

Silica sand with a density of 2587 kg/m^3^ and a water absorption of 1.24% was used for the production of mortars. The particle size distribution of the sand used is given in Figure 2. The scanning electron microscope (SEM) image of the silica sand is given in Figure 3. As is seen clearly from Figure 3, the sand particles are non-spherical and of an irregular shape but well-rounded with a smooth surface texture.

### 2.2. Mixture Proportions

The mixture proportions used in this study are given in Table 2. Five mixtures, a reference mixture without GP and four mixtures containing different amounts of GP, were prepared. NHL was used as a sole binder in the reference mixture. For the remainder, NHL was replaced with glass powder at different levels of 12.5%, 25.0%, 37.5%, and 50% by weight. The aggregate-to-binder ratio was 3.0 for all mixtures. The water-to-binder (W/B) ratio was 0.80 for all mixtures. By using the selected W/B ratio, 185 mm of flow diameter was achieved for the reference mortars to optimise strength, according to the recommendations of Hanley and Pavía [51]. Furthermore, the effect of GP on the consistency of mixtures were evaluated using the same W/B. For the identification of glass powder incorporated mortars, the abbreviation of glass powder (GP) and NHL replacement percentages were used. For instance, GP25 refers to mixtures in which NHL was replaced with 25% glass powder.

### 2.3. Mixture Preparation

A cement mixer was used for the production of mixtures in accordance with EN 196-1 [52]. First, powders and water were mixed for 30 s at low speed. During the subsequent 30 s of mixing, the sand was steadily added. Afterwards, the mixer was switched to high speed and further mixing was continued for another 30 s. While the mixture was resting for 90 s, the mixtures were scraped from the sides and paddled for 30 s. Finally, an additional 60 s of mixing at high speed was continued before casting. The total duration of mixing was 4 min. After mixing, the mixture was immediately cast into cubes with a dimension of 50 mm × 50 mm × 50 mm in two layers and each layer was tamped 25 times. Curing of the samples were carried out at three stages [17]. After casting, the moulds were placed in a sealed polyethylene bag for 2 days. Then, the samples were removed from the moulds and kept in the sealed polyethylene bag for another 5 days. Finally, samples were subjected to ambient curing in the laboratory at 20 ± 2 °C and relative humidity of 60 ± 5%, until testing age.

### 2.4. Methods

The flowability of the mortars was determined in accordance with EN 1015-3 [53]. The dry unit weight, porosity, and water absorption of samples were determined using Archimedes’ principle. The water absorptions by capillarity were measured on three samples according to EN 1015-18 [54]. The ultrasound pulse velocity of samples was determined prior to compressive strength tests in accordance with EN 12504-4 [55]. The compressive strengths of mortars were evaluated using a 250 kN-capacity universal testing machine, based on EN 1015-11 [56]. A fixed loading rate of 400 N/s was applied. For each mix, six 50 mm cubic samples were used. The reported results for compressive strengths are the mean value of six samples.

The carbonation depths of samples were determined after 28- and 91-day curing at a temperature of 21 ± 3 °C and a relative humidity of 60 ± 5%. Two cubic samples with a side of 5 cm were used. Samples were divided into two parts and 1% phenolphthalein alcohol solution was applied to the sections. Then, images of the cross-sections were captured with a high-resolution camera. The carbonation area was measured using Image J [57]. The carbonation percentages are presented by dividing the carbonation area by the sample area.

The microstructures of samples were investigated using scanning electron microscope (SEM) FEI, Quanta FEG 250. A small piece was taken from the inner parts of the samples after performing compressive strength tests. Afterwards, samples were oven dried at 40 °C for 24 h and coated with gold-palladium in a high-pressure vacuum prior to SEM.

## 3. Results and Discussion

### 3.1. Flow

**Figure 4:**
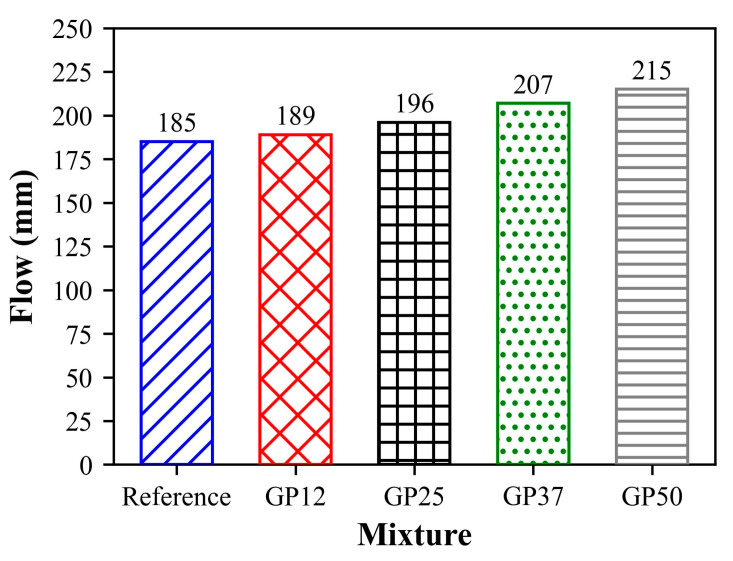
Flow of mixtures.

The flow test was conducted for the evaluation of the consistency of the mortars with the incorporation of the glass powder. The flow values of mortars were in the range between 185 mm and 210 mm as plotted in Figure 4.

The flow of mixtures increased gradually with the increase in GP content, indicating a decrease in the water demand. The main reasons for this result can be attributed to the coarser particle size of the glass powder than those of natural hydraulic lime, as well as the physical structure and low water absorption of glass particles. This finding can be related to the prior literature, in which the beneficial effect of glass powder on cement-based composites varies at different rates. Aliabdo et al. [58] stated that there is a 0.4% reduction in the water demand for each 5% of glass powder replacement in the glass powder incorporated cement pastes. Moreover, Islam et al. [59] reported a minor increase in the flow of cement-based mortars with up to 25% glass powder replacement. On the other hand, results conflict with some of the prior literature. Lu et al. [60] observed a reduction in the flow of cement-based mortars containing 20% glass powder, regardless of the fineness of GP used, the reason of this behaviour attributed to the particle size and irregular shape of the glass powders. Nahi et al. [61] stated that the effect of 10% and 20% glass powder replacement on the flow of cement-based mortars was negligible, but 35% and 60% glass powder replacement reduced flow slightly.

### 3.2. Carbonation Depth

**Figure 5:**
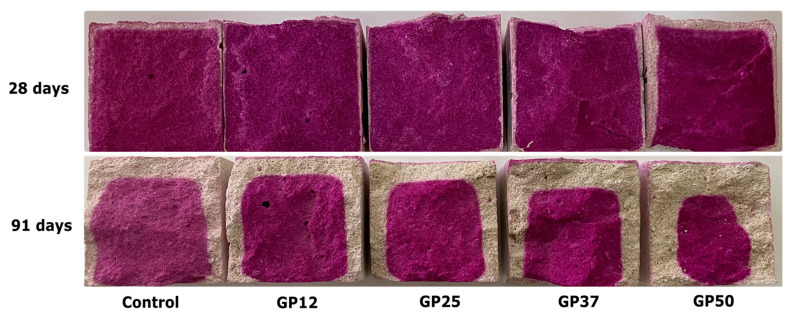
Cross-sections of samples after phenolphthalein indicator.

The carbonation depths of mortars with increasing glass powder content are presented in Figure 5. After 28 days of exposure, the carbonation percentage of the reference mixtures was about 16%. A significant reduction in carbonation percentage was observed with the replacement of NHL with 12.5% GP. However, a further increase in GP content in the mortars led to higher carbonation percentages. GP50 has the highest carbonation percentage of the samples at 28 days.

At 91 days, reference samples have the lowest carbonation percentage, about 38%. Contrary to the early ages, there is a gradual increase in carbonation percentages with GP incorporation at this age. A limited effect on carbonation was observed with up to 25% GP incorporation as a substitution of NHL at early ages. However, this behaviour has changed at later ages, where a dramatic increase in carbonation was observed. Furthermore, the carbonation percentage of the reference and GP12, and GP25, GP37 and GP50 after 91 days of curing were found to be about 150%, 800%, 650%, 370%, and 200% higher than their 28-day counterparts. The effect of glass powder on carbonation depth herein may be similar to the effect of nano-silica on the carbonation of NHL mortars, of which Luo et al. [62] observed that the incorporation of nano-silica at a level of up to 3% increased the carbonation percentage, suggesting their suitability for carbon dioxide collection and solidification.

### 3.3. Compressive Strength

**Figure 6:**
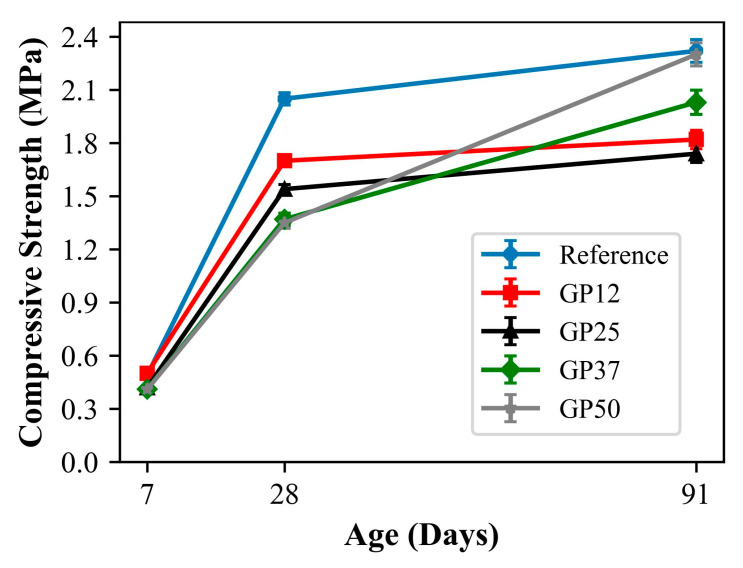
Compressive strength development of mortars.

**Figure 7:**
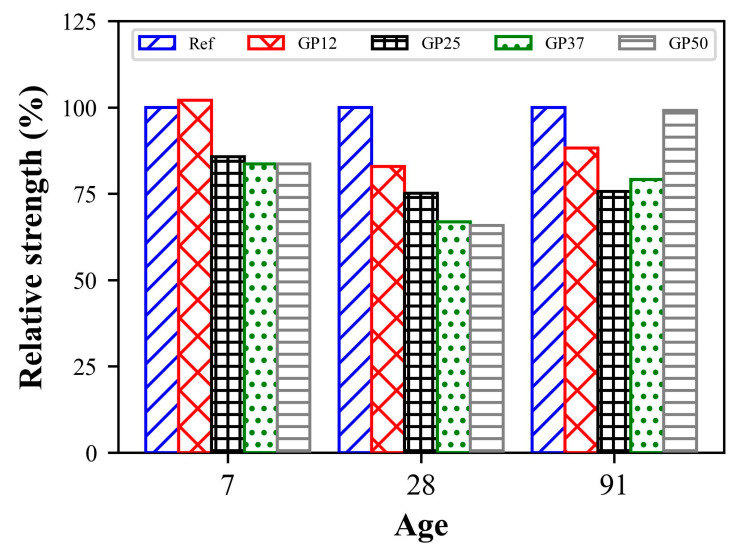
Relative strength of mortars.

The compressive strength development and relative strength of mortars up to 91 days are given in Figure 6 and Figure 7, respectively. At 7 days, the compressive strengths of the reference and GP12, and GP25, GP37 and GP50 were about 0.50 MPa and 0.41 MPa, respectively.

The strength of mortars containing 12.5% glass powder exhibited similar strength to those of the reference mortars. In the case of the replacement of hydraulic lime with more than 12.5% glass powder, the compressive strength of mortars decreased about 18%, indicating the negative effect of glass powder on early strength. It has been emphasised by many researchers that the contribution of glass powder to early age strength in inorganic binders is quite low [63]. The main reason for this behaviour is the insufficient amount of Ca(OH)_2_ reacting with reactive SiO_2_, which increases with high levels of glass powder incorporation [64].

At 28 days, the compressive strengths of mortars varied between 1.2 and 2.1 MPa. As it can be seen from the figure, the reference mortars containing no glass powder exhibited the highest strength and the lowest compressive strength was obtained by mortars with 50% glass powder. In addition, a reduction in strength is observed with the replacement of lime with glass powder for all mixtures. This reduction is about 82.9%, 75.1%, 66.8%, and 66.3% for hydraulic lime replacement levels of 12.5%, 25.0%, 37.5%, and 50%, respectively. This situation can be evaluated as an indication that the reaction of glass powder with hydraulic lime is not fully realised. Similar findings were reported in the prior literature. Edwards, Allen, Ball, and El-Turki [47] investigated the mechanical properties of NHL-based pastes incorporating up to 75% glass powder at 28 days. In the study, a reduction in compressive strength was reported with an increase in glass powder content, with the exception of pastes containing 10% glass powder which exhibited about 10% enhancement in compressive strength. Furthermore, Vyšvařil, Žižlavský, and Bayer [49] reported a gradual decrease in compressive strength with increasing glass powder in 28-day humid-cured NHL mortars.

At 91 days, the mortars revealed compressive strengths ranging from 1.74 to 2.31 MPa. The reference and GP50 exhibited the highest compressive strengths, which are about 22%, 25%, and 12% higher than GP12, GP25, and GP37, respectively. This indicates the enhancement of the pozzolanic reaction between NHL and GP at later ages. The results obtained differ from the results of Vyšvařil et al. [49], who reported improvements in the compressive strength of humid-cured NHL mortars containing up to 40% of glass powder. After the evaluation of compressive strength development of the mixtures, two different behaviours are observed at the early and later ages. The 7-day compressive strength of the reference and GP12, and GP25, GP37 and GP50 were found to be about 24%, 29%, 27%, 30%, and 20% of their 28-day compressive strength. On the other hand, the 91-day compressive strength of the reference and GP12, and GP25, GP37 and GP50 were found to be 13%, 6%, 13%, 48%, and 69% higher than their 28-day compressive strength. These results suggest that the strength development of NHL mortars over the curing duration increases with glass powder incorporation.

### 3.4. Unit Weight, Porosity, and Water Absorption

**Figure 8:**
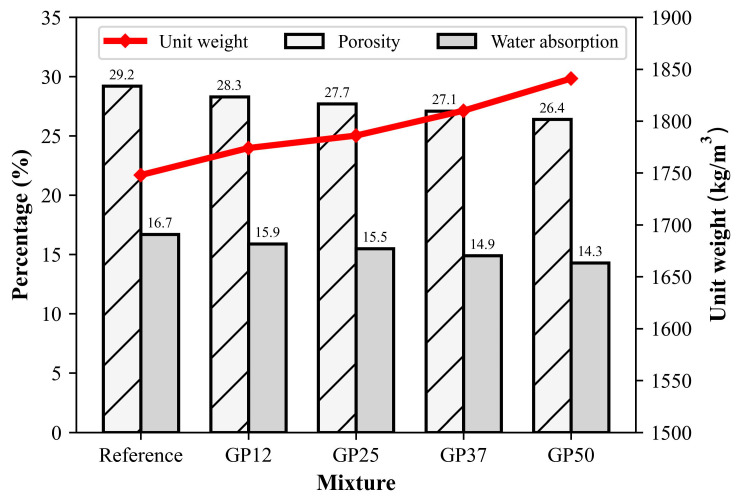
Unit weight, porosity, and water absorption of mortars at 28 days.

The dry unit weight, porosity, and water absorption of mortars are illustrated in Figure 8. The mortars revealed the dry unit weight ranged from 1759 to 1860 kg/m^3^. From the figure, it can be clearly seen that an explicit increase in dry unit weight was obtained with the incorporation of GP in comparison to the reference. The dry unit weights of mortars with 12.5%, 25.0%, 37.5%, and 50.0% glass powder were about 1.5%, 2.2%, 3.5%, and 5.3% higher than those of the reference mortar, respectively. The main reason for the observed increase in dry unit weight can be attributed to the enhanced compactability of mixtures with increasing consistency, despite the relatively similar densities of the powders employed.

The porosities and water absorptions of mortars ranged between 29.2% and 26.4%, and 16.7% and 14.3%, respectively. Reductions in the porosity and water absorption of the mortars were observed with the incorporation of GP. The reference mortars containing no GP exhibited the highest porosity and water absorption, indicating the presence of a high amount of water permeable pores. Furthermore, GP50 containing 50% glass powder had the lowest porosity and water absorption among all the mortars. These results may be attributed to the low water absorption characteristics of glass powder [65] and the formation of hydration products through pozzolanic reaction [66].

### 3.5. Ultrasonic Pulse Velocity (UPV)

**Figure 9:**
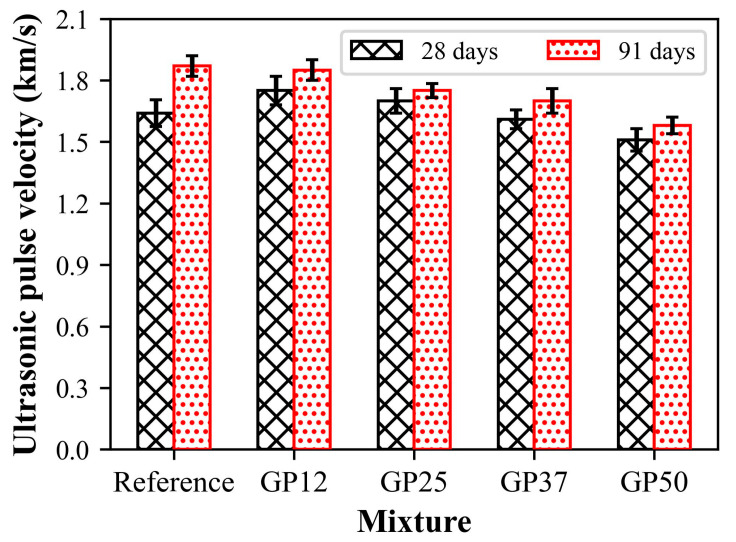
UPV results of mortars at different ages.

The measurement of quality and homogeneity of concrete using an ultrasonic pulse velocity test is very common and the preferred practice [67]. The ultrasonic pulse velocity results of the mortars measured at the age of 28 and 91 days before compressive strength tests are presented in Figure 9. The UPV of mortars increased with the increase in age regardless of the glass powder content of mortars. The increase in UPV values with the increase in curing duration from 28 days to 91 days was 13.6%, 5.9%, 3.1, and 5.3% for the reference, GP12, GP25, GP 37, and GP50, respectively. These results suggest the decrease in the porosity of mortars with increasing age, consistent with the prior literature. Moreover, this pattern in UPV with age can also be related to the compressive strength behaviour of mortars [68]. At 28 days, there is a slight increase, about 6%, in the UPV of mortars with the replacement of hydraulic lime with 12.5% glass powder. Further substitution of hydraulic lime with glass powder led to a substantial reduction in the UPV values of mortars. A similar trend was also seen in the UPV values of mortars at 91 days. The findings indicate that the incorporation of glass powder into the mixtures resulted in an increase in pore volume, which consequently led to a decrease in the UPV in comparison to the reference mortars.

### 3.6. Capillary Water Absorption

**Figure 10:**
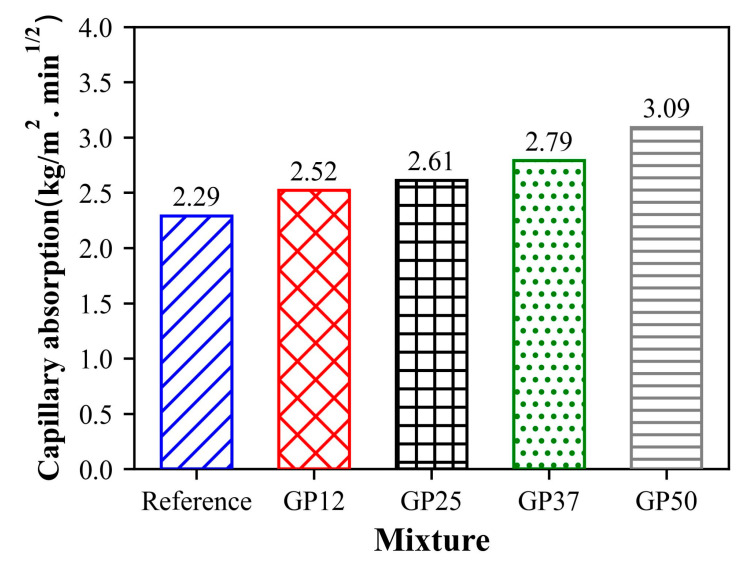
Water absorption coefficient by capillarity of mortars.

Figure 10 shows the capillary water absorption coefficient samples at 28 days. As can be observed from the figure, the reference samples exhibited the lowest capillarity coefficient, whereas the highest was obtained in the GP50 samples containing 50% of glass powder. A dramatic increase was observed in the capillary water absorption with increasing glass powder content in the mixtures. The increment of capillarity was about 10.1%, 14.4%, 21.8%, and 34.9% for the glass powder incorporation of 12.5%, 25%, 37.5%, and 50%, respectively. These results suggest that the increase in micropores leads to high capillary water absorption.

### 3.7. Microstructure

**Figure 11:**
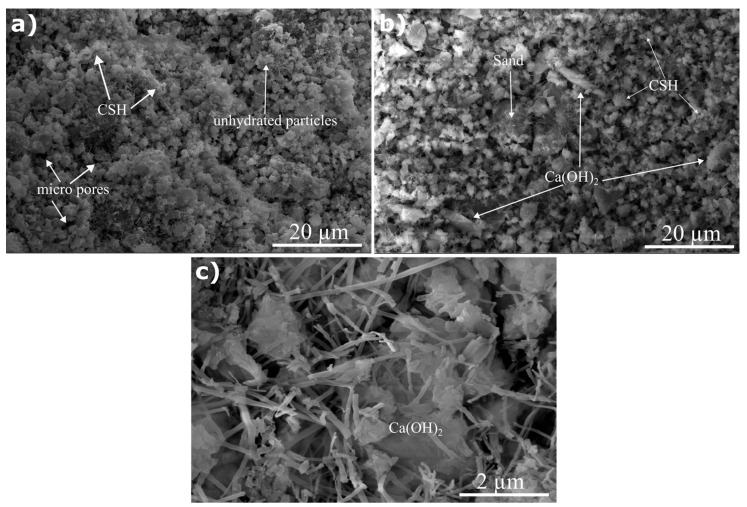
SEM images of samples: (**a**) Reference, (**b**) GP50 (Mag: 5000×), and (**c**) GP25 (Mag: 40,000×).

The influence of glass powder incorporation on the microstructure of mortars was analysed using the 91-day cured samples via SEM. Figure 11 presents the micrographs of the reference, GP50 and GP25. The difference in the microstructure of the samples can be seen explicitly at first glance. The reference sample exhibited the presence of amorphous calcium silicate gels (CSH) and voids within the structure (Figure 11a). Furthermore, unhydrated particles were observed. On the other hand, more pores and a larger porosity were observed on the structure of GP50 with clearly identifiable hexagonal Ca(OH)_2_ (Figure 11b). This result is also supported by the physical test results obtained. A detailed examination of the structure of GP25 revealed hexagonal Ca(OH)_2_, cubic CaCO_3_ crystals, and acicular crystals were present, which are also reported in the prior literature [69,70] (Figure 11c).

## 4. Conclusions

This study investigated the physical, mechanical, and microstructural properties of natural hydraulic lime mortars incorporating glass powder. With the replacement of natural hydraulic lime with glass powder, a notable decrease in water demand was observed. This reduction led to an increase in the consistency of mortars for a given water content, as a result of the higher unit weights obtained. The compressive strength of samples decreased with the increase in glass powder content in mortars for early and later ages. However, a noteworthy observation was observed in 91-day cured samples. Samples containing 50% glass powder exhibited compressive strengths comparable to the reference samples, indicating an enhancement of pozzolanic reaction in later ages. With the use of glass powder, changes in the void structure and amount affecting the physical properties were observed. While ultrasonic pulse velocity, porosity, and water absorption decreased with the substitution of hydraulic lime with glass powder, an increase in the amount of capillary water absorption was observed. The inclusion of glass powder exhibited a distinct microstructure, characterised by clearly identifiable calcium hydroxide particles, contrasting with the anhydrous particles and micropores observed in the reference sample. The findings imply that the incorporation of glass powder might offer economic and environmental advantages. Nevertheless, further research is necessary to ascertain the durability properties of glass powder incorporated hydraulic lime mortars in order to consider their practical applications.

## Figures and Tables

**Table 1:** Oxide composition of NHL and GP.

Oxide (%)	CaO	SiO_2_	Al_2_O_3_	Fe_2_O_3_	MgO	SO_3_	K_2_O	Na_2_O	L.O.I
NHL	63.94	9.12	2.40	0.98	2.34	0.97	0.59	0.24	17.0
GP	8.66	73.55	1.56	0.48	2.77	-	0.02	12.35	-

**Table 2:** Mixture proportions.

No	Mix ID	Hydraulic Lime	Glass Powder	Aggregate/Binder	Water/Binder
		(%)	(%)	Ratio	Ratio
1	Reference	100	-	3	0.80
2	GP12	87.5	12.5		
3	GP25	75.0	25.0		
4	GP37	67.5	37.5		
5	GP50	50.0	50.0		

## Data Availability

Dataset available on request from authors.
